# Brain structural network to investigate the mechanism of cognitive impairment in patients with acoustic neuroma

**DOI:** 10.3389/fnagi.2022.970159

**Published:** 2022-10-28

**Authors:** Xueyun Deng, Lihua Liu, Jiuhong Li, Hui Yao, Shuai He, Zhiwei Guo, Jiayu Sun, Wenke Liu, Xuhui Hui

**Affiliations:** ^1^Department of Neurosurgery, The Affiliated Nanchong Central Hospital of North Sichuan Medical College, Nanchong, China; ^2^Department of Neurosurgery, Southwest Hospital, Army Medical University, Chongqing, China; ^3^Department of Neurosurgery, West China Hospital of Sichuan University, Chengdu, China; ^4^Department of Geriatrics, The Affiliated Nanchong Central Hospital of North Sichuan Medical College, Nanchong, China; ^5^Department of Radiology, West China Hospital of Sichuan University, Chengdu, China; ^6^Department of Radiology, The Affiliated Nanchong Central Hospital of North Sichuan Medical College, Nanchong, China

**Keywords:** cognition, acoustic neuroma, vestibular schwannoma, diffusion tensor imaging, brain network

## Abstract

**Objective:**

Acoustic neuroma (AN) is a common benign tumor. Little is known of neuropsychological studies in patients with acoustic neuroma, especially cognitive neuropsychology, and the neuropsychological abnormalities of patients affect their life quality. The purpose of this study was to explore the changes in the cognitive function of patients with acoustic neuroma, and the possible mechanism of these changes by structural magnetic resonance imaging.

**Materials and methods:**

We used a neuropsychological assessment battery to assess cognitive function in 69 patients with acoustic neuroma and 70 healthy controls. Then, we used diffusion tensor imaging data to construct the structural brain network and calculate topological properties based on graph theory, and we studied the relation between the structural brain network and cognitive function. Moreover, three different subnetworks (short-range subnetwork, middle-range subnetwork, and long-range subnetwork) were constructed by the length of nerve fibers obtained from deterministic tracking. We studied the global and local efficiency of various subnetworks and analyzed the correlation between network metrics and cognitive function. Furthermore, connectome edge analysis directly assessed whether there were differences in the number of fibers in the different brain regions. We analyzed the relation between the differences and cognitive function.

**Results:**

Compared with the healthy controls, the general cognitive function, memory, executive function, attention, visual space executive ability, visual perception ability, movement speed, and information processing speed decreased significantly in patients with acoustic neuroma. A unilateral hearing loss due to a left acoustic neuroma had a greater impact on cognitive function. The results showed that changes in the global and local metrics, the efficiency of subnetworks, and cognitively-related fiber connections were associated with cognitive impairments in patients with acoustic neuroma.

**Conclusion:**

Patients exhibit cognitive impairments caused by the decline of the structure and function in some brain regions, and they also develop partial compensation after cognitive decline. Cognitive problems are frequent in patients with acoustic neuroma. Including neuropsychological aspects in the routine clinical evaluation and appropriate treatments may enhance the clinical management and improve their life quality.

## Introduction

Acoustic neuroma (AN), also known as vestibular schwannoma, is a benign tumor with a high incidence of 85% in the cerebellopontine angle, which mostly originates from the 8th cranial nerve sheath (Fortnum et al., 2009). About 90% of the patients present gradual progressive unilateral hearing loss (UHL)(Suzuki et al., 2010). AN is a common cause in UHL patients. UHL is defined as an asymmetric loss of hearing in which one side has a hearing loss and the other has a normal hearing (Vincent et al., 2015). The incidence of UHL is 7.2% in the United States (Golub et al., 2018). The difficulty of understanding and communicating in a noisy environment and the decreased ability of the sound source localization in patients with UHL, which affect the ability to receive important information from both sides simultaneously (Nelson et al., 2019).

Previous studies on patients with bilateral deafness have confirmed that the severity of hearing loss was positively correlated with cognitive impairment. Cognitive impairment in presbycusis with moderate and severe hearing loss is 1.4 and 1.6 times higher than in healthy people, respectively (Davies et al., 2017). Presbycusis is related to the increased risk of cognitive impairment, including decreased executive function (Gurgel et al., 2014), memory (Lin et al., 2011), and psychomotor processing disorders (Chen et al., 2018). Early intervention of the hearing can improve the cognitive function and behavior state in bilateral presbycusis patients (Ma et al., 2016; Adrait et al., 2017). Studies have shown that a partial or unilateral decrease in hearing in patients with UHL, which can affect the central auditory cortex and remodel vision and other sensory systems (Propst et al., 2010), even cognitively-related brain regions (Zhang et al., 2018), resulting in the decline in advanced cognitive functions such as memory, language, and learning. Children with UHL have poorer language development and cognition than normal children, and they have problems such as language retardation and inattention (Carew et al., 2018; van Wieringen et al., 2019), among whom the proportion of children with behavioral problems (25%) is higher than the normal children (Lieu et al., 2012). The connections in the brain network of executive function, language comprehension, and cognition in children with UHL are different from those of normal children (Jung et al., 2017).

To date, there are few reports on whether the hearing loss in UHL, especially AN patients, will cause cognitive dysfunction; the conclusions from previous studies were different; the number of reported cases was low; and little was known about the mechanism. In this study, we analyzed the cognitive function between AN patients and healthy controls (HCs) using various neuropsychological tests. Furthermore, we first systematically explored the possible underlying mechanism of cognitive impairment in AN patients using diffusion MRI. We constructed the structural brain network based on diffusion tensor imaging (DTI) data, and analyzed the network based on graph theory and other related analyses of brain networks, in order to study the brain connectomics of cognitive dysfunction in AN patients.

## Materials and methods

### Participants

Sixty-nine right-handed AN patients (44 females and 25 males, age range: 19–76 years) were recruited from the outpatient and ward of Neurosurgery of West China Hospital of Sichuan University, from October 2019 to July 2020. Seventy right-handed hearing controls (48 females and 22 males, age range: 26–74 years) were also enrolled in our study. The demographic information for these subjects is shown in Table 1; Supplementary Tables 2, 3. The experiment was approved by the hospital ethics committee, and all participants signed the informed consent.

**Table 1:** Comparison of clinical data and cognitive function among LAN, RAN, and HC groups.

	LAN (*n* = 44)	RAN (*n* = 25)	HC (*n* = 70)	F/H/T values	*p* value	*Post-hoc*
gender (male)	19 (43.2%)	6 (24.0%)	22 (31.4%)	2.979	0.225^a^	N/A
age (yrs)	50.28 ± 13.58	50.26 ± 12.49	46.54 ± 10.05	1.769	0.174^b^	N/A
years of education (yr)	11.00 (7.30)	9.00 (9.00)	9.00 (7.00)	3.790	0.150^c^	N/A
Course of disease (yr)	2.00 (4.17)	2.50 (4.50)	N/A	−1.111	0.267^d^	N/A
THI	14.00 (6.00)	12.00 (8.00)	N/A	−0.131	0.896^d^	N/A
Left PTA (dB HL)	55.73 ± 26.14	22.15 ± 10.67	N/A	5.186	<0.001^e*^	N/A
Right PTA (dB HL)	17.50 (10.00)	66.74 ± 34.06	N/A	−4.784	<0.001^d*^	N/A
tumor diameter (cm)	3.02 ± 1.21	3.06 ± 1.13	N/A	−0.138	0.891^e^	N/A
MoCA scores	21.00 (6.00)	20.00 (9.00)	25.50 (4.00)	38.792	<0.001^c*^	RAN<HC LAN < HC
RAVLT immediate recall	34.00 (16.00)	31.00 (15.00)	47.00 (19.00)	32.651	<0.001^c*^	RAN<HC LAN < HC
RAVLT delay recall	6.00 (5.00)	5.00 (4.00)	9.00 (5.00)	18.441	<0.001^c*^	RAN<HC LAN < HC
Stroop A (s)	32.00 (27.00)	36.00 (16.75)	27.00 (14.50)	5.714	0.057	N/A
Stroop B (s)	49.00 (32.00)	53.00 (24.00)	38.00 (20.00)	15.631	<0.001^c*^	RAN>HC LAN > HC
Stroop C (s)	129.00 (83.00)	131.00 (64.00)	85.50 (53.00)	24.963	<0.001^c*^	RAN>HC LAN > HC
SDMT	39.00 (28.00)	34.00 (34.00)	45.00 (28.00)	10.824	0.004 ^c*^	RAN<HC LAN < HC
TMT A (s)	52.00 (65.00)	67.00 (63.00)	40.50 (30.00)	18.100	<0.001^c*^	RAN>HC LAN > HC
TMT B (s)	170.00 (193.00)	230.00 (191.00)	104.00 (116.00)	15.320	<0.001^c*^	RAN>HC LAN > HC
HAMD	9.00 (7.00)	10.00 (5.00)	2.00 (3.00)	69.242	<0.001^c*^	RAN>HC LAN > HC
HAMA	6.00 (6.00)	7.00 (6.00)	2.00 (2.00)	62.254	<0.001^c*^	RAN>HC LAN > HC

^a^*p* and ^b^*p* values were obtained by the R × C chi-square test and ANOVA test, respectively. ^c^*p* and ^d^*p* values were obtained by Kruskal-Wallis and Mann–Whitney (nonparametric test), ^e^*p* values were obtained by t-test. *F* values, H values, and T values were obtained by ANOVA, Kruskal-Wallis, and t-test. All data were expressed as mean ± SD, median (interquartile range), or number (percentage). The significance level was set at *p* < 0.05. *p < 0.05. LAN: left acoustic neuroma; RAN: right acoustic neuroma; HC: healthy controls; PTA: pure tone average; THI: tinnitus handicap inventory; N/A: not available. HAMD/ HAMA.

The inclusion criteria of AN were as follows: (1) unoperated unilateral acoustic neuroma; (2) no history of mental or neurological diseases; (3) right-handedness; (4) no contraindications of MRI (such as spatial claustrophobia and metal implants). Healthy control (HC) group: (1) normal hearing; (2) right-handedness; (3) no history of craniocerebral trauma; (4) 18–75 years old.

Exclusion criteria were as follows: (1) previous diseases such as chronic otitis media that affect hearing threshold; (2) excluding the previous history of ear surgery, hearing loss caused by ototoxic drugs, or wearing hearing aids; (3) bilateral acoustic neuroma, such as neurofibromatosis type 2; (4) conductive hearing loss; (5) craniocerebral trauma; (6) tumors with other intracranial sites and nature; (7) excluding the history of craniocerebral surgery; (8) those who could not cooperate to complete the neuropsychological test (including daltonism and color weakness).

The average air conduction thresholds at four frequencies (0.5, 1, 2, and 4 kHz) were calculated as the pure tone average (PTA), representing the hearing levels of the subjects. According to the World Health Organization, hearing loss was classified as mild (PTA 26-40 dB HL), moderate (PTA 41–60 dB HL), severe (PTA 61–80 dB HL), profound (PTA > 81 dB HL).

Patients with acoustic neuroma are often complicated with tinnitus symptoms. In this study, AN patients accompanying tinnitus were assessed using the tinnitus handicap inventory (THI) scale (Newman et al., 1996). Higher scores indicate greater severity and greater impact on daily life.

Cognitive functions of all subjects were assessed, including the following measures: Montreal cognitive assessment (MoCA), Rey auditory verbal learning test (RAVLT) immediate memory and delayed memory, Stroop color-word test A, B, and C (Stroop A, B, and C), symbol digit modalities test (SDMT), Trail-Making Test A and B (TMT A and B), Hamilton depression scale (HAMD), and Hamilton anxiety scale (HAMA).

### MRI data acquisition

The data acquisition parameters were as follows: DTI and 3D high-resolution T1WI were acquired on GE 750 W 3.0Tesla magnetic resonance equipment (General Electric Medical System, Milwaukee, WI, United States) with a 32-channel head coil. Acquisition parameters used for DTI sequence were as follows: TR = 7,000 ms, TE = 72 ms, FOV = 24 cm × 24 cm，acquisition matrix = 256 × 256，flip angle = 90°，slice thickness / slice spacing = 4.0 mm/0.0 mm (no intervals). A total of 34 slices were scanned. Diffusion-sensitive gradients were applied in 51 directions (b = 1,000 s/mm^2^) and one b0 (b = 0 s/mm^2^) images. T1 scanning parameters were as follows: slice thickness = 1 mm, scanning matrix = 512 × 512, voxel size = 0.5 × 0.5 × 1.0 mm^3^.

### Data preprocessing

The MRI data preprocessing and network construction were performed using PANDA (www.nitrc.org/projects/panda; Cui et al., 2013). The main steps were as follows: data quality check, data format conversion, and head eddy-current effect correction. The whole brain fiber bundle was tracked using deterministic fiber tracking (Mori et al., 1999). The main results tracked by PANDA software were fiber number (FN) and fiber length (FL). The next steps were to explore the possible mechanism of cognitive function changes in patients with AN from different perspectives based on these results. Firstly, the structural brain network was constructed using the fiber number, and we analyzed the network properties and the correlation between the network properties and cognition function. Secondly, we directly compared the FN in any two brain regions between AN patients and healthy controls. Thirdly, the FL was used to construct short-range, middle-range, and long-range subnetworks, and the relationships between subnetwork topological properties and cognition decline were discussed.

### Network construction and graph theory metrics calculation

The FN was calculated by the deterministic fiber tracking and was registered to the individual Anatomical Automatic Labeling 90 regions (AAL90). Due to tumors compressing the cerebellum and brainstem in some patients, it may affect the study of the cerebellum, so we constructed networks based on the 90 cerebral regions of the AAL atlas, excluding the cerebellar regions. The FN value of every two brain regions constructed a weighted matrix. The connections were considered effectively structurally connected if at least three fibers in two brain regions in 80% of the subjects (Shu et al., 2015), transforming the weighting matrix into a binary matrix. Network topological properties were calculated using the GRETNA toolbox (http://www.nitrc.org/projects/gretna/), including global network metrics (Shu et al., 2015; Wang et al., 2015a): 1. small world property: shortest path length (Lp), clustering coefficient (Cp), and small-world parameters (λ, γ, and σ). 2. global efficiency (E_g_), local efficiency (E_loc_); local metrics: node local efficiency, node efficiency, node shortest path, node clustering coefficient, and degree centrality (see Supplementary Table 1).

Meanwhile, for the weighted FN matrix, we compared the FN between 90 × 90 brain regions using network-based statistic (NBS) multiple comparison correction (Zalesky et al., 2010). The NBS procedure was performed as follows: (1) admit weighted connections with statistical analysis surpassing *p <* 0.001 between groups; (2) seek clusters that suprathreshold connections; and (3) *via* permutation testing (5,000 permutations), compute *p* values (FWE-corrected) for each cluster. Significant differences between groups were performed using *p <* 0.05 to control the FWE after NBS correction.

The FL was classified according to the following criteria: 1. short-range subnetwork: fiber length less than 40 mm 2. middle-range subnetwork: fiber length 40-80 mm; 3. long-range subnetwork: fiber length was greater than 80 mm (Wang et al., 2013). The global and local efficiency of these subnetworks were calculated, respectively. And Spearman correlation was assessed between the network metrics and the cognitive scale.

### Statistical analysis

SPSS (version 23.0, IBM, United States) was used for statistical analysis. Data are presented as mean ± standard deviation for normally distributed variables or median (interquartile range) for not normally distributed variables. Categorical variables were expressed as percentage. When comparing two groups of independent data, t-test (two-tailed) was used if the data were satisfied normal distribution with homogeneous variance, otherwise, Mann–Whitney U nonparametric test was performed. According to the comparison among the two subgroups of AN (LAN&RAN) and the HC groups, one-way ANOVA or Kruskal-Wallis H test was used to test whether the data were satisfied normal distribution and homogeneous variance. The qualitative data were compared by χ ^2^ test. Spearman correlation analysis was used to explore the relationship between clinical data and cognitive function. The statistically significant *p* value was less than 0.05.

The global metrics of the brain network were statistically analyzed by using the SPSS23.0 and the GRETNA software (http://www.nitrc.org/projects/gretna/; Wang et al., 2015b) was used for statistical analysis for local metrics. The comparison of local metrics is *p* < 0.05, using a False Discovery Rate (FDR) for multiple comparison correction.

## Results

### Demographic characteristics

A total of 139 subjects were included in this study, including 69 patients with AN (LAN: RAN = 44: 25) and 70 HC patients. No significant differences in gender, age, and years of education between the two groups or among the left acoustic neuroma (LAN), right acoustic neuroma (RAN), and HC groups were attested (*p* > 0.05), as reported in Table 1, Supplementary Table 2. There were no significant differences in the course of the disease, PTA on the affected side, and THI scores between the LAN and RAN groups, as displayed in Table 1.

### Comparison of cognitive function and correlation analysis between AN and HC groups

**Figure 1:**
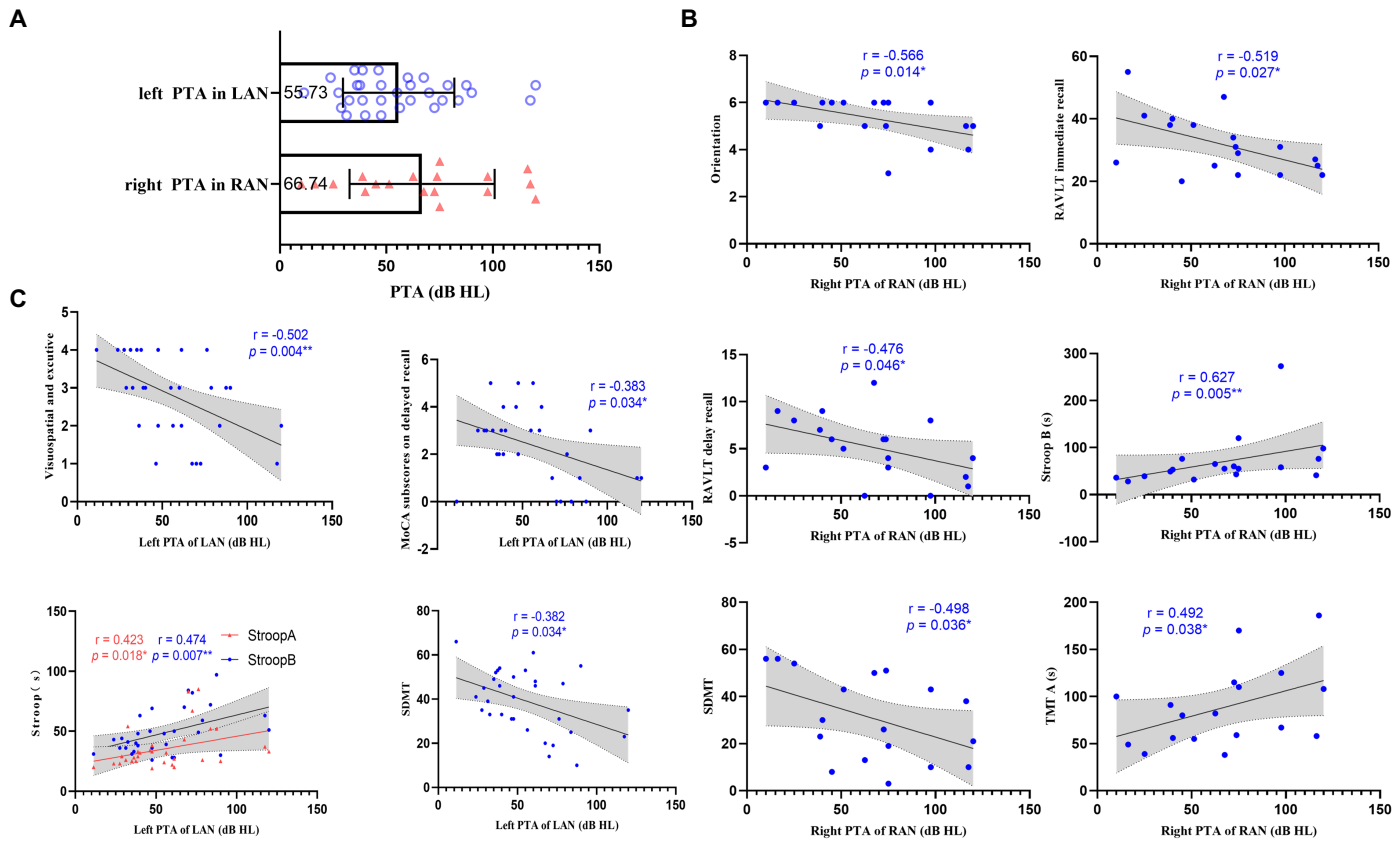
Relation between pure tone average (PTA) and cognition. **(A)** Scatter plots representing the distribution of PTA in the left acoustic neuroma (LAN) and right acoustic neuroma (RAN) groups. The numbers in the box represent the average PTA of the affected side in LAN and RAN patients, respectively. **(B)** Scatterplots showing the associations between right PTA and cognitive scale in the RAN patients. **(C)** Scatterplots showing the correlations between left PTA and cognitive scale in the LAN patients. The correlation analysis between PTA and cognition shows that hearing loss in AN patients affects cognitive functions such as visuospatial executive, memory, attention, motor speed, visual perception, and executive control. Error bars in **(A)** represent mean ± standard deviation. The gray regions in **(B,C)** show a 95% confidence interval. *, *p* < 0.05;**, *p* < 0.01.

Compared with the HC group, the patients with AN performed worse in MoCA, RAVLT, Stroop, SDMT, and TMT (*p* < 0.05; See Supplementary Tables 2, 3), and both groups (LAN and RAN) also performed worse in MoCA, RAVLT, Stroop B, C, SDMT, and TMT (*p* < 0.05; see Table 1; Supplementary Table 4). Spearman correlation analysis showed that the left-sided PTA of patients with LAN was negatively correlated with MoCA subscores on visuospatial executive and delayed recall, and SDMT, and positively correlated with Stroop A and B. In RAN patients, the right-sided PTA was negatively correlated with MoCA subscores on orientation, RAVLT, and SDMT, while positively correlated with Stroop B and TMT-A. See Figure 1.

### Comparison of cognitive function among patients with different grades of AN and HC groups

According to Koos et al., (1993), all AN patients were graded into four groups: grade 1 (tumor diameter < 1 cm) in 1 cases; grade 2 (tumor diameter 1-2 cm) in 16 cases; grade 3 (tumor diameter 2–3 cm) in 17 cases; grade 4 (tumor diameter > 3 cm) in 35 cases. Because the number of patients with grade 1 was too small to analyze statistically, the cognitive functions of patients of other grades were compared with those of the HC group. The results showed that compared with the HC group, the cognitive function of patients with grades 2–4 decreased, as listed in Supplementary Tables 5, 6. To investigate whether tumor size affects cognitive function, tumor size was defined on the basis of the longest diameter, and Spearman correlation analysis was performed between tumor diameter and cognitive function, and no significant correlation was found between tumor size and cognitive function.

### Comparison of cognitive function among patients with different degrees of hearing loss and HCs

According to WHO grade (1997), the hearing of the affected side (tumor side) of the patients with AN were as follows: normal hearing (PTA < 25 dB HL) in 5 cases, mild loss (PTA 26–40 dB HL) in 12 cases, moderate loss (PTA 41–60 dB HL) in 9 cases, severe loss (PTA 61–80 dB HL) in 13 cases, profound loss (PTA > 81 dB HL) in 10 cases. Compared with the HC group, the cognitive function of AN patients with mild to profound hearing loss decreased to various degrees, see Supplementary Tables 7, 8.

### Results of global metrics

#### Small world property

**Figure 2:**
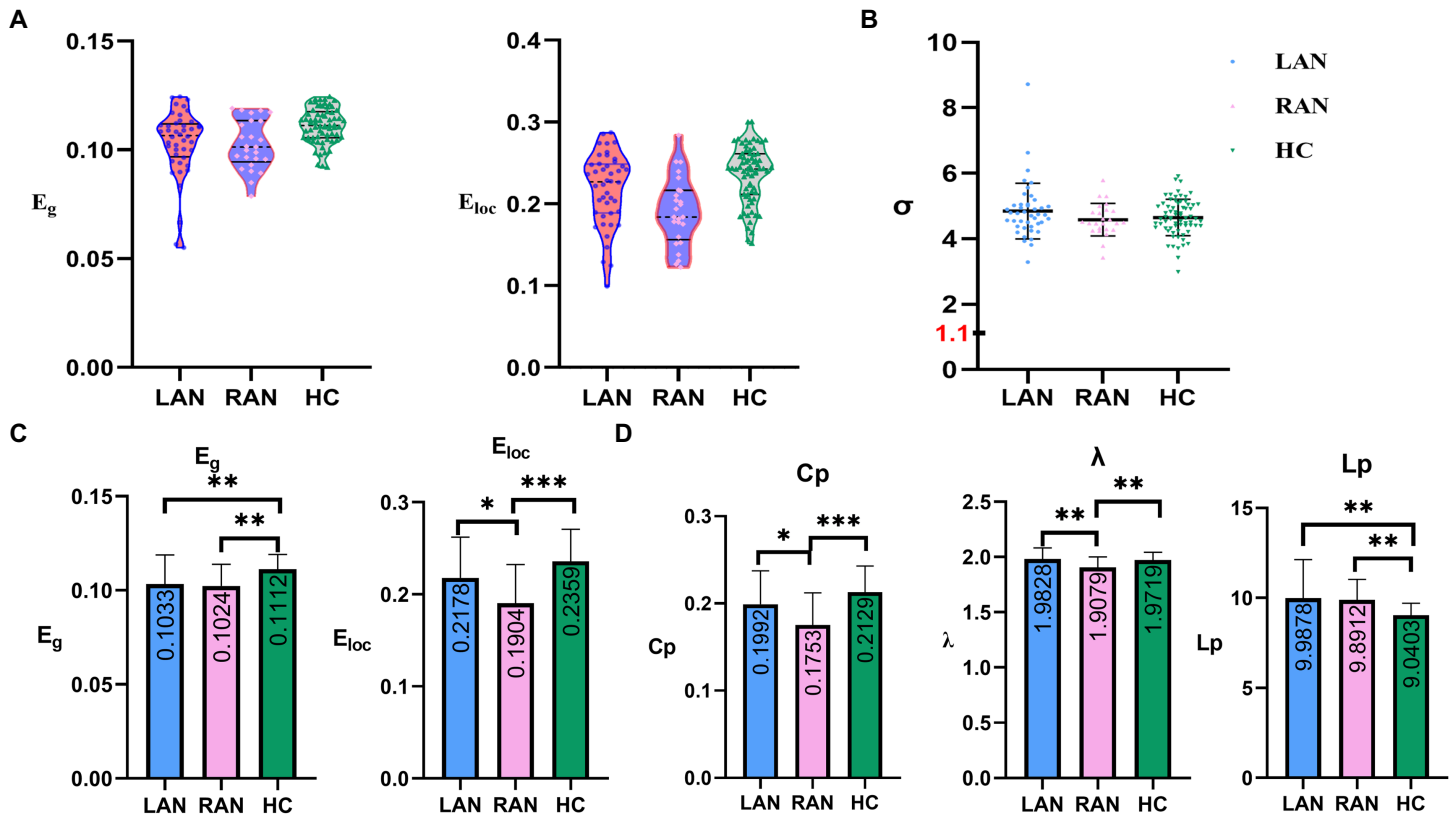
The comparisons of global metrics in three groups. **(A)** Scatter plots representing the distribution of global efficiency (E_g_) and local efficiency (E_loc_) in LAN, RAN, and HC groups. **(B)** Scatter plots representing the distribution of small world properties (σ) in the LAN, RAN, and HC groups. **(C)** E_g_ and E_loc_ of structural brain network for three groups. E_g_ and E_loc_ in AN patients decreased compared to the HC group. **(D)** Shortest path length (Lp), clustering coefficient (Cp), and small-world parameters λ (λ = Lp_real_ / Lp_random,_ Lp_real_: the Lp of the real network; Lp_random_: the Lp of random network) of structural brain network for three groups. The numbers in the box represent mean values. Error bars represent mean ± standard deviation.

The small world property with LAN was tested by one-sample t-test, the same for RAN. The results were *p* < 0.001, showing that both left and right AN patients have obvious “small world property.” No significant differences were found among the LAN, RAN, and HC groups. The small-world networks can highly effectively integrate, segregate, and transmit information with low cost. The above results indicated that patients with auditory neuroma still have the characteristics of high efficacy and less energy loss. Compared with the HC group, the Cp and λ were significantly lower in RAN patients (*p* < 0.001, *p* = 0.002, respectively), and the Lp was significantly higher in LAN patients (*p* = 0.003). See Figure 2.

Global efficiency (E_g_) and local efficiency (E_loc_).

Compared with the HC group, the E_g_ of the LAN and RAN groups decreased (*p* = 0.009, 0.003, respectively); the E_loc_ of the RAN group decreased significantly (*p* < 0.001). The E_loc_ of the RAN group was lower than that of the LAN group (*p* = 0.036). See Figure 2.

### Results of local metrics

**Figure 3:**
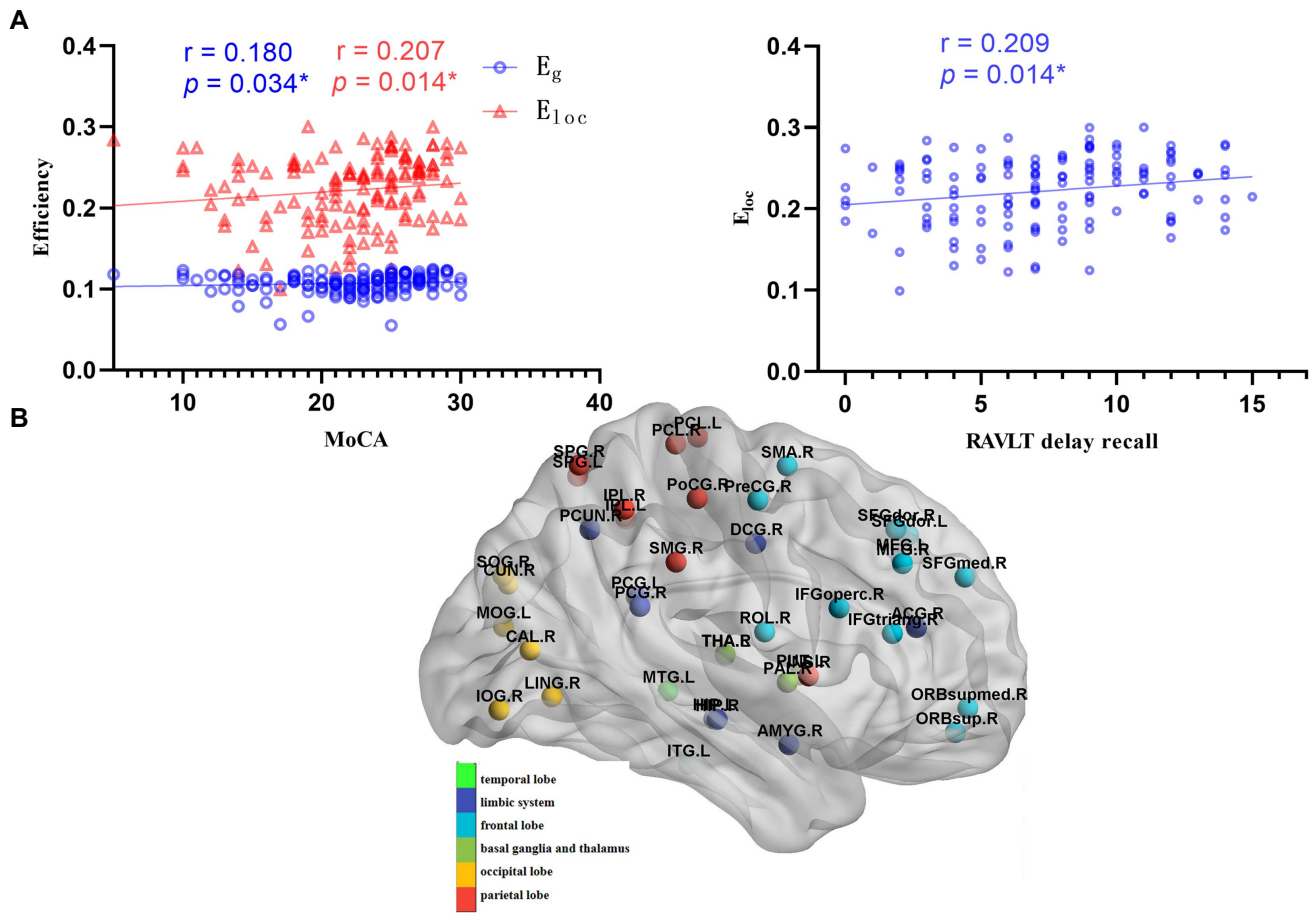
Correlations between global metrics and cognition. **(A)** Correlations between global metrics (E_g_ and E_loc_) and cognitive scales (MOCA and RAVLT delay recall); Statistically significant Spearman correlation coefficients (*n* = 139, *p <* 0.05) are shown (MOCA: Montreal cognitive assessment; RAVLT: Rey auditory verbal learning test). **(B)** Schematic diagram of the differential node efficiency among the LAN, RAN, and HC groups. AN patients showed a more extensive decrease of node efficiency, mainly in the frontal lobe, occipital lobe, parietal lobe, limbic system, basal ganglia, thalamus.

The results of local metrics were as follows:

Compared with the node efficiency of the HC group, both left and right AN patients showed a more extensive decrease, mainly in the frontal lobe, occipital lobe, parietal lobe, limbic system, basal ganglia, thalamus, and so on. Only in the LAN group, the node efficiency of the left middle and inferior temporal gyrus increase, which was associated with compensation for auditory deprivation. The differential brain regions of node efficiency are shown in Figure 3B; Supplementary Tables 8, 9. Furthermore, compared with the HC group, all the other local metrics decreased, such as node local efficiency, node shortest path, node clustering coefficient, and degree centrality, see Supplementary information (*p* < 0.05, FDR corrected).

### Correlations between graph theory metrics and cognitive performance

The results showed that the E_g_ and E_loc_ were positively correlated with the MoCA (*r* = 0.180, *p* = 0.034; *r* = 0.207, *p* = 0.014, respectively), and the E_loc_ was positively correlated with RAVLT delayed recall (*r* = 0.209, *p* = 0.014), as shown in Figure 3A.

The node efficiency of 90 brain regions can reflect the inherent property of each brain region to some extent, so Spearman correlation analysis was performed between the node efficiency of differential brain regions and cognitive performance. Widespread decreased node efficiency affected general cognitive function mainly in the frontal lobe, parietal lobe, insular, and limbic system, involving the default mode network (DMN), frontoparietal network, and salience network, which related to advanced cognition. The results are shown in Supplementary Tables 9, 10.

### Results of connectome edge analysis

**Figure 4:**
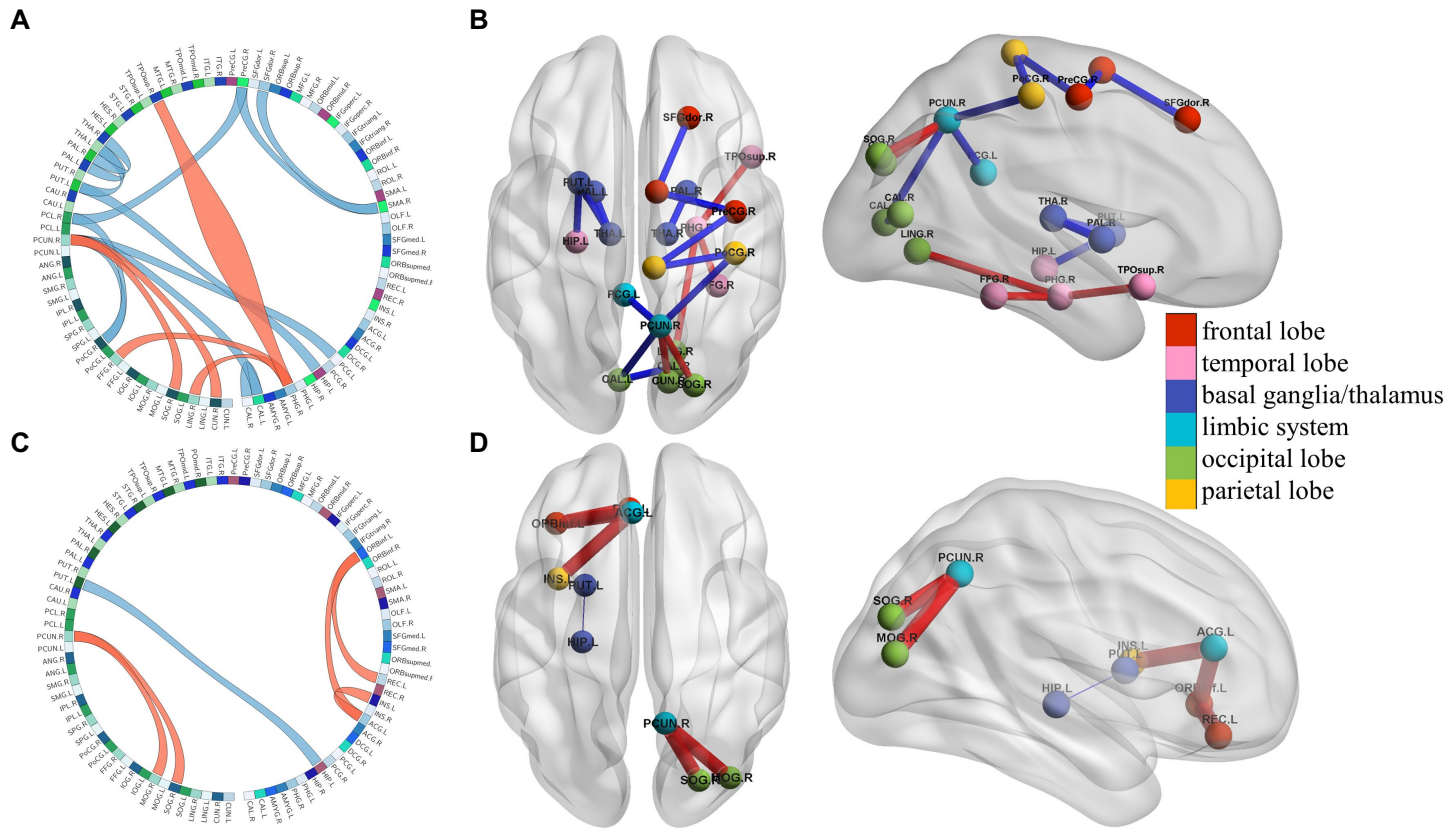
The results of connectome edge analysis. Ninety brain regions constitute the circle part of the circos diagram. Red lines represent the increase in the number of white matter fibers compared to the HC group, which are defined as positive connections, and blue lines represent the decrease in the number of fibers, which are defined as negative connections. The red and blue lines have the same meaning in **(A–D)**, **(A,B)** Significant differences in the number of white matter fibers in 90 brain regions between LAN and HC groups are shown. The brain regions of negative connections mainly involve the default mode network, sensorimotor network, dorsolateral prefrontal loop, basal ganglia and thalamus, and medial temporal lobe memory (MTL) system, and are related to cognition declines such as memory, language, abstraction, and visuospatial ability. The positive connections mainly involve brain regions between the precuneus and occipital lobe fibers and memory-related fiber connections, suggesting that the body compensates for hearing loss and cognitive decline. (**C**,**D**) Significant differences between RAN and HC groups are shown. Fibers between the left lenticular putamen and the left hippocampus were the only negative connection in RAN patients. The fibers belonging to the MTL system are associated with memory. Positive connection regions mainly involve the occipital lobe, the frontostriatal circuits, the salience network, and the MTL system, and are associated with the visual system, attention, cognition switching, and memory, revealing that the body presents anatomical fiber connection enhancement to compensate for cognitive declines such as attention and memory.

Using connectome edge analysis, fiber number in the brain region between AN and HC groups was directly compared. We defined positive or negative connections according to the increase or decrease in the number of fibers between brain regions. We found that compared with the HC group, the LAN and RAN patients had both positive and negative connections. See Figure 4 for details.

### E_g_ and E_loc_ of the three subnetworks

**Figure 5:**
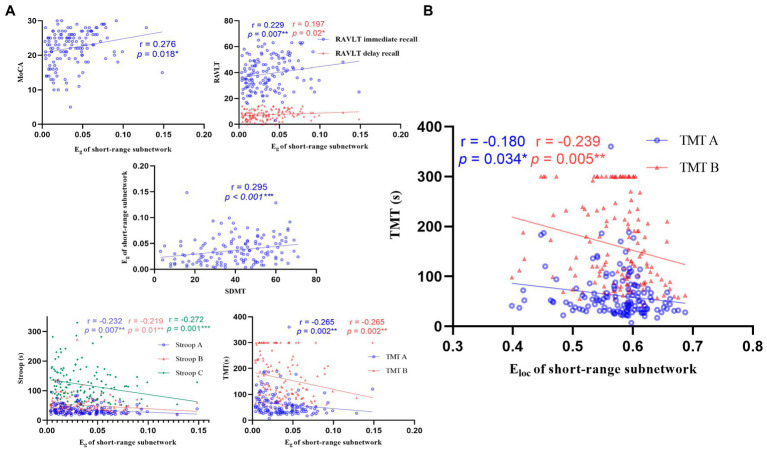
Correlations between the global metrics (E_g_, E_loc_) of short-range subnetwork and cognitive scale. **(A)** Correlations between the E_g_ of short-range subnetwork and cognitive scale. The E_g_ of short-range subnetwork was negatively correlated with cognition function (*n* = 139, *p* < 0.05). **(B)** Correlations between the E_loc_ of short-range subnetwork and TMT. MOCA: Montreal cognitive assessment; RAVLT: Rey auditory verbal learning test; SDMT: symbol digit modalities test; Stroop: Stroop color-word test; TMT: trail-making test.

Compared with the HC group, the E_g_ of the LAN group decreased significantly in the short-range subnetwork (*p* = 0.002), middle-range subnetwork (*p* < 0.001), and long-range subnetwork (*p* = 0.001), and the E_loc_ of LAN patients in the short-range subnetwork decreased significantly (*p* = 0.002). There was no significant change in RAN patients. See Figure 5.

### Correlation analysis between metrics of subnetworks and cognitive scale

The E_g_ of short-range and middle-range subnetwork was positively correlated with MoCA, RAVLT, and SDMT, and negatively correlated with Stroop and TMT. The E_g_ of the long-range subnetwork and the E_loc_ of the short-range subnetwork were negatively correlated with Stroop C and TMT, respectively. See Figure 5; Supplementary Figure 3.

## Discussion

### The cognitive performance in AN patients

The LAN and RAN groups performed worse on MoCA, RAVLT, Stroop, SDMT, and TMT tests than those of the HC group, revealing that both LAN and RAN patients developed cognitive dysfunction such as memory, attention, executive function, movement speed, and information processing speed. Previous studies have established that UHL children are more likely to develop cognition dysfunction such as hypoprosexia, language development retardation, and poor learning. Goebel and Mehdorn (2018) enrolled 27 patients with AN and 18 patients with posterior fossa meningioma, and the authors found that most of the patients (69%) develop cognitive impairment, the most common performance is attention (such as alertness) and visual movement speed, which is consistent with our findings. Fan et al., (2020) investigated 25 LAN and 15 RAN patients and demonstrated that the general cognitive function is normal in the AN group, but the attention, information processing efficiency, executive function, and memory decreased. Our conclusion is inconsistent with theirs. We found that general cognitive function, attention, executive function, memory, visuospatial and perception, movement speed, and information processing speed in AN patients were significantly lower than those of normal people. In our study, the AN patients performed worse in more neuropsychological tests, maybe because our sample was larger and the statistical efficiency was relatively efficient. The course of the disease was negatively correlated with cognition, showing that with the development of the course of the disease, the ability of visuospatial executive, attention, memory, motor speed, and information processing speed decrease gradually, so early intervention may delay the effect of disease on cognitive function (Supplementary Figures 1, 2). We found that patients with different grades of AN had different degrees of cognitive decline, however, there was no correlation between tumor size and cognitive function, demonstrating that tumor size was not the main factor for cognitive decline in AN patients. Meanwhile, this study also found that cognitive function decreased in patients with different degrees of hearing loss, and there was a significant correlation between PTA and cognitive function. It is suggested that hearing loss may have a greater effect on the cognitive function of patients with AN. Better hearing protection or reconstruction, may be better cognitive function.

### The relation between global metrics and cognitive function

We found that AN patients had small-world properties, however, the average shortest path was higher than that of normal people. The E_g_ was lower than that of normal people, and the E_loc_ decreased in RAN patients. The small-world networks can highly effectively integrate, segregate, and transmit information with low cost (Bullmore and Sporns 2012). Generally, the fundament of brain organization efficiently is two basic principles: functional separation and integration (Tononi et al., 1994; Sporns 2011). The clustering coefficient and E_loc_ reflect the functional separation; the average shortest path and E_g_ reflect the functional integration. In our study, the clustering coefficient, the average shortest path, E_g_, and E_loc_ changed, suggesting that functional separation and integration decreased in AN patients. The correlation analysis between global and local efficiency and cognitive scale in AN patients showed that global and local efficiency (E_g_, E_loc_) were positively correlated with MoCA, and local efficiency (E_loc_) was also positively correlated with RAVLT delayed recall. The decrease in the global and local efficiency of patients led to general cognitive impairment, and the decrease in delayed recall ability was related to the decrease in local efficiency of the brain network. The global efficiency reflects the ability of the brain network to transmit information and the degree of network polymerization, and the local efficiency is used to measure the efficiency of distributed information processing in the whole brain structure network. The decrease in global and local efficiency in AN patients led to the decline of whole brain information transmission ability and information processing efficiency, which may be the reason for cognitive impairment.

### The relation between local metrics and cognitive function

The brain regions with decreased node efficiency affected MoCA (representing general cognitive function) mainly in the frontal lobe, parietal lobe, insular, and limbic system, involving the default model network (DMN), frontoparietal network, salience network which are related to advanced cognition. We first found that the decline of immediate and delayed recall in RAVLT was mainly related to the decrease of node efficiency in the right supramarginal gyrus, dorsolateral superior frontal gyrus, opercular part of inferior frontal gyrus, rolandic operculum, insula, precentral gyrus, postcentral gyrus, superior parietal gyrus, cuneus, angular gyrus, and precuneus (Supplementary Tables 9, 10). The process of memory includes encoding and retrieval, and the memory retrieval brain region includes the right frontal lobe, anterior cingulate cortex, mid-parietal regions, and thalamus (Nyberg et al., 1996). Tulving et al., (1994) found that the left frontal lobe is more active in memory encoding and the right frontal lobe in memory retrieval, which is called HERA model by authors. We also found the brain regions related to RAVLT immediate and delayed recall were mainly located in the frontal lobe, insular, and parietal lobe. Smith et al., (1991) demonstrated that the visuospatial components of working memory are significantly activated in the right hemisphere, including the prefrontal lobe, premotor area, parietal lobe, and occipital lobe. In this study, the brain regions related to immediate memory were also located in the prefrontal lobe, premotor area, and parietal lobe, which was consistent with the literature. The decline in memory in AN patients was related to the decrease of node efficiency in brain regions involved in memory retrieval and working memory.

### Connectome edge analyses and cognitive function

Previous studies have supported that the right parahippocampal gyrus, temporal pole, and fusiform gyrus are involved in the composition of memory (Aminoff et al., 2013). We had confirmed that decreased memory occurred in LAN patients in our study. The fiber connections between the right precuneus and the occipital lobe were strengthened, indicating that the compensation of the visual pathway increased after left auditory deprivation in LAN patients. The negative connections in LAN patients were as follows: (1) Right precuneus and left calcarine cortex, left posterior cingulate gyrus, postcentral gyrus. The precuneus and posterior cingulate gyrus are the key brain regions of the DMN, and their functions include memorizing, making social inferences, and looking forward to the future (Buckner and DiNicola 2019). The internal connections of DMN in LAN patients were weakened, which may affect the corresponding cognitive function. (2) Right precentral gyrus and supplementary motor area, dorsolateral superior frontal gyrus; right paracentral lobule and precentral gyrus and postcentral gyrus. These brain regions belong to the somatosensory motor system. Studies have affirmed that the sensorimotor system initiates and regulates the sensation and movement of the body, and plays an important role in speech processing such as vocabulary, phonetics, sentences, and chapter processing (Fischer and Zwaan 2006). During phonological processing, hearing syllables can activate the motor cortex corresponding to the mouth and tongue related to pronunciation (Pulvermüller et al., 2006). When understanding certain movements, the premotor area or motor cortex can be specifically activated (Shtyrov et al., 2014). Activations in the sensorimotor area increase during reading action-related texts (Speer et al., 2005). Therefore, we speculate that the decrease in sensorimotor network connections may lead to the decline of language and abstract ability in LAN patients. (3) Right thalamus and pallidum; left thalamus and pallidum, putamen. The fibers which involve projection fibers from the dorsolateral prefrontal lobe loop originate from the frontal lobe, pass through the caudate nucleus, pallidum, thalamus, and final project to the dorsolateral area of the frontal lobe. The function is to mediate executive function, and executive dysfunction is observed after injury (Grahn et al., 2009). The decline of executive function in AN patients may be associated with the negative connections in this loop. The subthalamic globus pallidus and globus pallidus subthalamic fibers are the afferent and efferent fibers of the globus pallidus, respectively, belonging to the basal ganglion nucleus. Numerous studies have confirmed that basal ganglia lesions may lead to cognitive function, including decreased memory (Grahn et al., 2009), visuospatial impairment (Su et al., 2007), and so on. (4) Left putamen and left hippocampus. The fibers belong to the medial temporal lobe system and are involved in memory (Sekeres et al., 2018).

The positive connections in RAN patients were as follows: (1) Right precuneus and right superior and middle occipital gyrus. The enhanced connections may indicate that the activation of visual fibers in RAN patients compensates for auditory deprivation. (2) Left orbital part of inferior frontal gyrus and anterior cingulate and paracingulate gyri, gyrus rectus. The fibers involve the frontostriatal circuits, including the orbitofrontal gyrus, anterior cingulate gyrus, striatum, ventromedial prefrontal lobe, and other nerve loops, which are mainly related to attention function (Fan et al., 2005). The enhancement of the connections between the left orbitofrontal and anterior cingulate gyrus may be linked to compensation of function after attention dysfunction in RAN patients. (3) Left anterior cingulate and paracingulate gyri and insular. Both regions are key nodes of the salience network (Seeley et al., 2007), which mainly plays a key role in the switching between cognitive function-related networks (Sidlauskaite et al., 2016). In RAN patients, the connections between the left anterior cingulate gyrus and insular were enhanced, suggesting that patients may need additional resources to maintain attention to the outside world after auditory deprivation. Fibers between the left lenticular putamen and the left hippocampus were the only negative connection in RAN patients. The fibers belonging to the medial temporal lobe system are associated with memory (Lisman et al., 2017). The negative connection may play a role in memory dysfunction in RAN patients.

### The study of subnetworks and cognitive function

**Figure 6:**
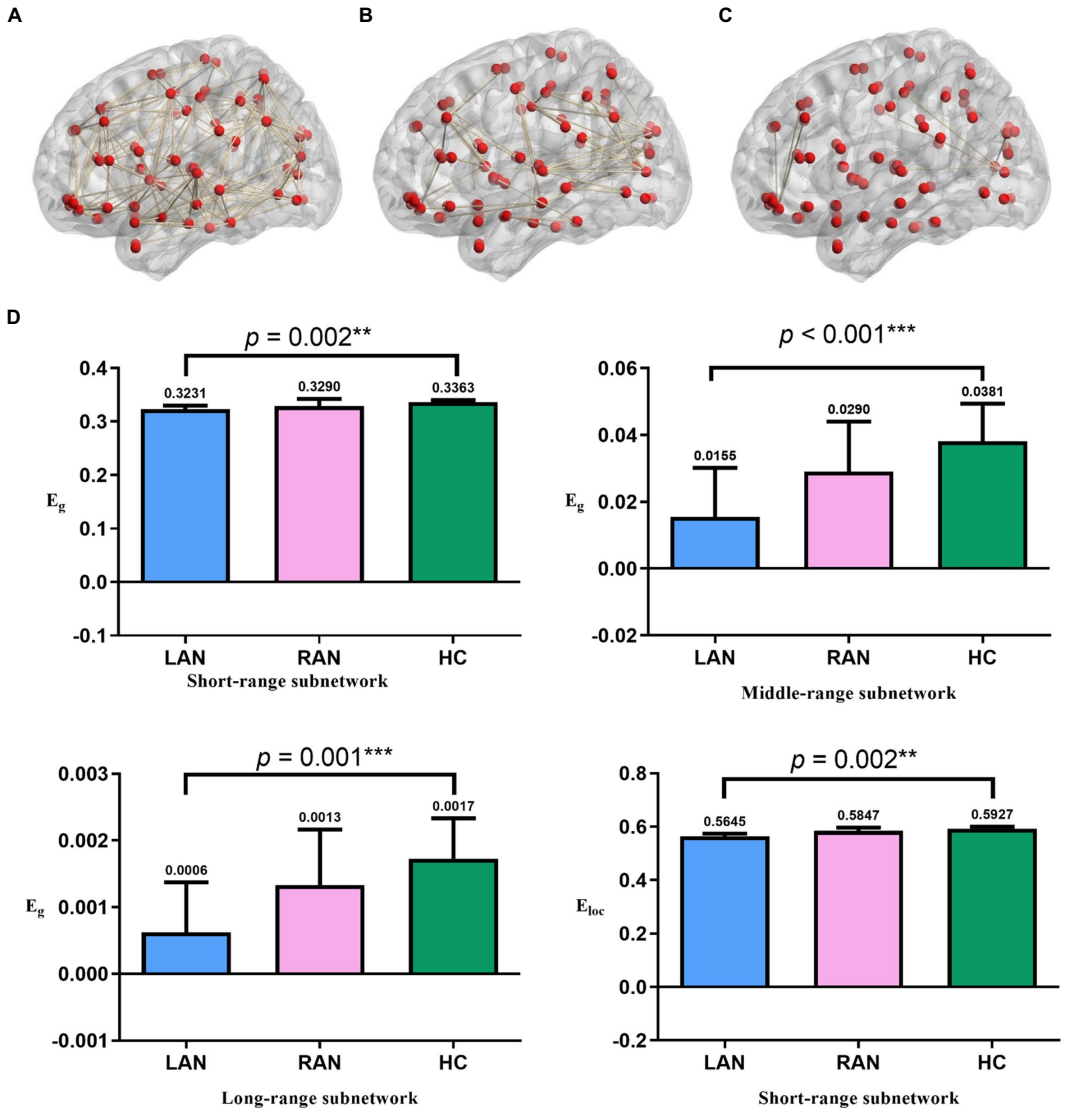
The results of subnetworks. **(A)** Short-range subnetwork of one subject **(B)** middle-range subnetwork of the same subject. **(C)** long-range subnetwork of the same subject. **(D)** Comparison of global metrics (E_g_ and E_loc_) in the three groups. The global metrics of the three subnetworks in AN patients significantly decreased. The numbers above the error bars represent median values. Error bars represent median ± 95%CI. * *p* < 0.05, ** *p* ≤ 0.01, *** *p* ≤ 0.001; LAN: left acoustic neuroma; RAN: right acoustic neuroma; HC: health control.

This study found that the E_g_ of the three subnetworks and the E_loc_ of the short-range subnetwork decreased in LAN patients, showing a decline in the ability of information integration and transmission in LAN patients, while no significant changes in RAN patients (Figure 6). The short-range subnetwork is usually located in the same brain region and reflects changes in local brain regions, and the middle-range subnetwork is distributed in the same functional region, while the long-range subnetwork connects different brain regions and is mainly responsible for information transmission (Su et al., 2015). These subnetworks ensure the separation and integration of brain functions. The subnetworks had changed in AN patients, affecting information transmission in the brain. In LAN patients, the E_g_ of the three subnetworks and the E_loc_ of the short-range subnetwork decreased, resulting in a decline in the ability of brain information transmission and integration.

In this study, we found that the effects of LAN and RAN on the brain may be different. The LAN tends to decrease in structure and function, while the RAN tends to be compensated in structure and function, which is more resistant to hearing loss and more stable. The LAN patients may be more sensitive to auditory deprivation, and the RAN patients may tend to be functionally compensated. The RAN mainly affected the left dominant hemisphere and may have a stronger compensatory mechanism, so the RAN had less impact on patients and the performance was more stable. This was consistent with the literature (Wang et al., 2014; Zhang et al., 2015).

The shortcomings of this study are as follows: 1. This study is a cross-sectional study, which failed to follow up on the changes in cognitive function with the development of disease course and hearing level. However, the correlation analysis part provides a gradual decline in cognitive function with the progress of the disease course. 2. This study failed to follow up on whether the cognitive function of patients with acoustic neuroma recovered after hearing improvement.

## Conclusion

In summary, in this study, we found that the cognitive function decreased in AN patients, and first systematically studied its possible mechanism. The results showed that widespread changes occurred in the cognition-related structural and functional regions of AN patients. The global efficiency and local efficiency of the structural brain network decrease in AN patients, which is closely related to cognitive dysfunction. In this study, using the edge analysis of brain structural networks in AN patients for the first time, we found that AN affected the fiber connections between cognitive-related brain regions. The global and local efficiency of subnetworks based on fiber length decreased, which affected patients’ general cognition, memory, execution, attention, processing speed, and visuospatial ability. The mechanisms of the left and right acoustic neuromas affecting the brain network are different. The LAN tends to decrease in structure and function, while the RAN tends to be compensated in structure and function, which is more resistant to hearing loss and more stable. Cognitive problems are frequent in AN patients. Including neuropsychological aspects in the routine clinical evaluation and appropriate treatment may enhance clinical management and improve the quality of life of patients.

## Data availability statement

The raw data supporting the conclusions of this article will be made available by the authors, without undue reservation.

## Ethics statement

The studies involving human participants were reviewed and approved by Ethics Committee of Nanchong Central Hospital. The patients/participants provided their written informed consent to participate in this study. Written informed consent was obtained from the individual(s) for the publication of any potentially identifiable images or data included in this article.

## Author contributions

XD, XH, and LL designed the study. XD, JL, and HY collected the data. XD, SH, JS, and ZG analyzed the data. XD wrote the paper. WL, LL, and XH drafted the paper. All authors contributed to the article and approved the submitted version.

## Funding

This work was supported by Bureau of Science and Technology Nanchong city (grant no. 19SXHZ0273 and 20YFZJ0115), Nanchong Social Science Federation (grant no.NC21B188), Sichuan Province Medical Youth Innovative Research Project Program (grant no. Q21029), Primary Health Development Research Center of Sichuan Province (grant no. SWFZ20-C-069), and Special Funding for Postdoctoral Research Projects of Chongqing (grant no. 2021XM3012). Open access publication fees received from Nanchong Central Hospital.

## Conflict of interest

The authors declare that the research was conducted in the absence of any commercial or financial relationships that could be construed as a potential conflict of interest.

## Publisher’s note

All claims expressed in this article are solely those of the authors and do not necessarily represent those of their affiliated organizations, or those of the publisher, the editors and the reviewers. Any product that may be evaluated in this article, or claim that may be made by its manufacturer, is not guaranteed or endorsed by the publisher.

## Abbreviation

AN: Acoustic neuroma
DMN: Default ode network
DTI: Diffusion tensor imaging
E_g_: Global efficiency
E_loc_: Local efficiency
FN: Fiber number
FL: Fiber length
HC: Healthy control
LAN: Left acoustic neuroma
MoCA: Montreal cognitive assessment
PTA: Pure tone average
RAN: Right acoustic neuroma
RAVLT: Rey Auditory Verbal Learning Test
SDMT: Symbol-digital modalities test
TMT: Trail-making test
UHL: Unilateral hearing loss.
