# Supplementary material for: Brain structural network to investigate the mechanism of cognitive impairment in patients with acoustic neuroma
Source: Front Aging Neurosci. 2022 Oct 28;14:970159. doi: 10.3389/fnagi.2022.970159 (PMC9650538; doi:10.3389/fnagi.2022.970159)
Supplement: Supplementary file 1 [file Data_Sheet_1.docx]

**Supplementary_material**

**Brain Structural Network to investigate the mechanism of cognitive impairment in patients with acoustic neuroma**

**Table 1 Main parameters of brain network topology properties**

| Parameters of  brain network | | Calculation Formula | Remarks |
| --- | --- | --- | --- |
| Shortest path length (Lp) | 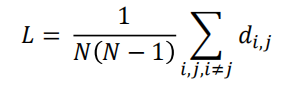 | | The Lp reflects the efficiency of information communication. The shorter the Lp is, the higher the efficiency of information communication is, and the less resources are needed. |
| Global efficiency (E_g_) | 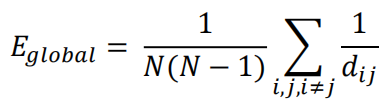 | | E_g_ reflects the information transmission capability between nodes in the network |
| Local efficiency  (E_loc_) | | 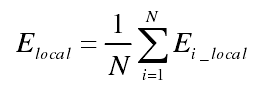 | E_loc_ measures the efficiency of distributed information processing in networks of the brain. |
| Clustering coefficient (Cp) | | 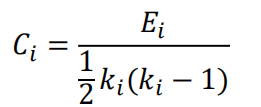 | Cp describes the local structural properties. The higher the Cp, the higher the local efficiency and the faster the local information transmission. |
| Degree centrality（Dc） | | 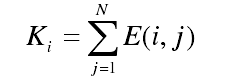 | Dc measures the importance of a single node in the network. |

**1. Comparison of cognitive function and mental status between the AN and HC groups**

Compared with the HC group, the patients with AN performed worse in MoCA, RAVLT, Stroop, SDMT, and TMT (*P* < 0.05). Especially, there were significant differences in MoCA score, RAVLT, Stroop C, SDMT, TMT between the AN and HC groups (*P* < 0.001). The scores of HAMD and HAMA in the AN group were significantly higher than the HC group (*P* < 0.001). See Table 2, Table 3 and Table 4.

**Table 2 Demographic and clinical characteristics of AN and HC**

|  | AN(n=69) | HC(n=70) | Z / T values | *P* |
| --- | --- | --- | --- | --- |
| gender(male) | 25(36.2%) | 22(31.4%) | 0.358 | 0.549^a^ |
| age(yr) | 50.28±13.14 | 46.54±10.05 | 1.883 | 0.062^b^ |
| years of education(yr) | 9.62(6.50) | 10.38(7.00) | -0.671 | 0.502^c^ |
| Course of disease(yr) | 2.00(3.59) | N/A | N/A | N/A |
| Left PTA (dB HL) | 43.39±27.11 | N/A | N/A | N/A |
| Right PTA (dB HL) | 35.43±32.36 | N/A | N/A | N/A |
| THI | 13.00(7.00) | N/A | N/A | N/A |
| MoCA | 21.00(8.00) | 25.50(4.00) | -6.089 | <0.001^c*^ |
| RAVLT immediate recall | 34.00(16.00) | 47.00(19.00) | -5.705 | <0.001^c*^ |
| RAVLT delay recall | 5.68±3.35 | 9.04±3.29 | -4.103 | <0.001^b*^ |
| Stroop A(s) | 32.00(21.50) | 27.00(14.50) | -2.387 | 0.017^c*^ |
| Stroop B(s) | 49.00(30.00) | 38.00(20.00) | -3.879 | <0.001^c*^ |
| Stroop C(s) | 130.50(78.00) | 85.50(53.00) | -4.994 | <0.001^c*^ |
| SDMT | 36.00(31.00) | 45.00(28.00) | -3.242 | <0.001^c*^ |
| TMT A(s) | 58.00(68.00) | 40.50(30.00) | -4.169 | <0.001^c*^ |
| TMT B(s) | 180.00(191.00) | 104.00(116.00) | -3.890 | <0.001^c*^ |
| HAMD | 9.00(6.00) | 2.00(3.00) | -8.247 | <0.001^c*^ |
| HAMA | 7.00(6.00) | 2.00(2.00) | -7.864 | <0.001^c*^ |

^a^*P* and ^b^*P* were obtained by chi-square test and t-test (two-tailed), respectively. ^c^*P* obtained by Mann-Whitney U nonparametric test. Z values and T values were obtained by nonparametric test and t-test. All data are presented as mean ± SD, median (interquartile range), or number (percentage). The significance level was set at *P* < 0.05. **P* <0.05. AN: acoustic neuroma. HC: healthy controls. PTA: pure tone average; N/A: not available.

**Table 3 Analysis of the effects of AN and HC on MoCA**

|  | AN（n=69） | HC（n=70） | Z values | *P* |
| --- | --- | --- | --- | --- |
| visuospatial executive | 3.00（1.00） | 4.00（2.00） | -6.374 | <0.001^*^ |
| naming | 3.00（2.00） | 3.00（1.00） | -1.920 | 0.055 |
| attention | 5.00（1.00） | 6.00（0.00） | -5.140 | <0.001^*^ |
| language | 1.00（1.00） | 2.00（1.00） | -4.112 | <0.001^*^ |
| language: Sentence repetition | 0.00（1.00） | 1.00（1.00） | -3.575 | <0.001^*^ |
| language: fluency task | 1.00（0.00） | 1.00（0.00） | -3.901 | <0.001^*^ |
| abstract thinking | 1.00（2.00） | 2.00（1.00） | -3.082 | 0.002^*^ |
| delayed recall | 2.00（2.00） | 3.00（3.00） | -3.953 | <0.001^*^ |
| orientation | 6.00（1.00） | 6.00（0.00） | -3.601 | <0.001^*^ |
| MoCA scores | 21.00（8.00） | 25.50（4.00） | -6.089 | <0.001^*^ |

Z and *P* values were obtained by Mann-Whitney U nonparametric test. Data are presented as median (interquartile range). * *P* <0.05。AN: acoustic neuroma. HC: healthy controls.

**Table 4 Comparison of MoCA among LAN, RAN, and HC groups**

|  | LAN(*n =* 44） | RAN(*n =* 25） | HC(*n =* 70） | H values | *P* | Post-hoc |
| --- | --- | --- | --- | --- | --- | --- |
| visuospatial executive | 3.00(2.00） | 2.00(1.00） | 4.00(2.00） | 41.421 | <0.001^*^ | RAN<HC  LAN<HC |
| naming | 3.00(1.00） | 2.00(2.00） | 3.00(1.00） | 8.552 | <0.001^*^ | RAN<HC |
| attention | 5.00(1.00） | 5.00(1.00） | 6.00(0.00） | 26.700 | <0.001^*^ | RAN<HC  LAN<HC |
| language | 1.50(1.00） | 1.00(1.00） | 2.00(1.00） | 18.038 | <0.001^*^ | RAN<HC  LAN<HC |
| language: Sentence repetition | 1.00(1.00） | 0.00(1.00） | 1.00(1.00） | 14.450 | 0.001^*^ | RAN<HC  LAN<HC |
| language: fluency task | 1.00(0.00） | 1.00(0.00） | 1.00(0.00） | 15.241 | <0.001^*^ | RAN<HC  LAN<HC |
| abstract thinking | 1.00(2.00） | 1.00(2.00） | 2.00(1.00） | 9.947 | 0.007^*^ | RAN<HC  LAN<HC |
| delayed recall | 2.00(2.00） | 2.00(2.00） | 3.00(3.00） | 15.843 | <0.001^*^ | RAN<HC  LAN<HC |
| orientation | 6.00(1.00） | 6.00(1.00） | 6.00(0.00） | 13.461 | 0.001^*^ | RAN<HC  LAN<HC |
| MoCA scores | 21.00(6.00） | 20.00(9.00） | 25.50(4.00） | 38.792 | <0.001^*^ | RAN<HC  LAN<HC |

*P* and H values were obtained by Kruskal-Wallis H (nonparametric test). Data were expressed as median (interquartile range). * *P* < 0.05。LAN: left acoustic neuroma; RAN: right acoustic neuroma; HC: healthy controls; MoCA: Montreal cognitive assessment.

**2. Comparison of cognitive function among patients with different** **grades of AN and HC group**

According to Koos grade^1^, All AN patients were graded into four groups: grade 1 (tumor diameter < 1cm) in 1 cases; grade 2 (tumor diameter 1-2cm) in 16 cases; grade 3 (tumor diameter 2-3cm) in 17 cases; grade 4 (tumor diameter > 3cm) in 35 cases. Because the number of patients with grade 1 was too small to analyze statistically, the cognitive functions of patients of other grades were compared with those of the HC group. The results showed that compared with the HC group, the cognitive function of patients with grade 2-4 decreased, as listed in Table 5 and Table 6.

|  | Grade 1 (tumor diameter < 1cm) (n=1) | Grade 2 (tumor diameter 1-2cm) (n=16) | Grade 3 (tumor  diameter 2-3cm)  (n=17) | Grade 4 (tumor diameter > 3cm) (n=35) |
| --- | --- | --- | --- | --- |
| visuospatial executive | N/A | -2.919(0.004)^**^ | -4.371(*P* < 0.001)^***^ | -5.552(*P* < 0.001)^***^ |
| naming | N/A | -0.039(0.969) | -1.422(0.155) | -2.355(0.019)^*^ |
| attention | N/A | -3.366(0.001) | -3.322(0.001)^***^ | -4.576(*P* < 0.001)^***^ |
| language | N/A | -1.100(0.271) | -3.778(*P* < 0.001)^***^ | -3.429(0.001)^***^ |
| language: Sentence repetition | N/A | -0.692(0.489) | -3.324(0.001)^***^ | -3.048(0.002)^**^ |
| language: fluency task | N/A | -2.951(0.003)^**^ | -4.061(*P* < 0.001)^***^ | -3.680(*P* < 0.001)^***^ |
| abstract thinking | N/A | -0.123(0.902) | -2.081(0.037)^*^ | -3.381(0.001)^***^ |
| delayed recall | N/A | -2.483(0.013)^*^ | -1.804(0.071) | -3.512(*P* < 0.001)^***^ |
| orientation | N/A | -2.642(0.008)^**^ | -2.560(0.010)^**^ | -3.327(0.001)^***^ |
| MoCA scores | N/A | -2.652(0.008)^**^ | -3.846(*P* < 0.001)^***^ | -5.527(*P* < 0.001)^***^ |

**Table 5 Comparison of MoCA among patients with different grades of AN and HC group**

Data are presented as Z values (*P* values). Z and *P* values were obtained by Mann-Whitney U nonparametric test. *, *P* < 0.05; **, *P* ≤ 0.01; ***, *P* ≤ 0.001; N/A: not available; MoCA: Montreal cognitive assessment.

|  | Grade 1 (tumor diameter < 1cm)  (n = 1) | Grade 2 (tumor diameter 1-2cm)  (n = 16) | Grade 3 (tumor diameter 2-3cm)  (n = 17) | Grade 4 (tumor diameter > 3cm)  (n = 35) |
| --- | --- | --- | --- | --- |
| MoCA scores | N/A | -2.652(0.008)^**^ | -3.846(*P* < 0.001)^***^ | -5.527(*P* < 0.001)^***^ |
| RAVLT immediate recall | N/A | -2.804(0.005)^**^ | -3.476(0.001)^***^ | -5.044(*P* < 0.001)^***^ |
| RAVLT delay recall | N/A | -1.966(0.053) | -3.457(0.001)^***^ | -6.035(*P* < 0.001)^***^ |
| Stroop A (s) | N/A | -0.626(0.531) | -1.746(0.081) | -2.905(0.004)^**^ |
| Stroop B (s) | N/A | -0.183(0.855) | -2.758(0.006)^**^ | -4.199(*P* < 0.001)^***^ |
| Stroop C (s) | N/A | -0.921(0.357) | -3.630(*P* < 0.001)^***^ | -4.993(*P* < 0.001)^***^ |
| SDMT | N/A | -0.807(0.419) | -2.560(0.010)^**^ | -3.794(*P* < 0.001)^***^ |
| TMT A (s) | N/A | -1.410(0.159) | -2.597(0.009)^**^ | -3.978(*P* < 0.001)^***^ |
| TMT B (s) | N/A | -0.339(0.735) | -2.003(0.045)^*^ | -4.570(*P* < 0.001)^***^ |

**Table 6 Comparison of cognitive function among patients with different grades of AN and HC group**

Data are presented as Z values (*P* values). Z and p values were obtained by Mann-Whitney U nonparametric test. *, *P* < 0.05; **, *P* ≤ 0.01; ***, *P* ≤ 0.001. N/A: not available.

**3.** **Comparison of cognitive function among patients with different degrees of hearing loss and HCs.**

According to WHO grade (1997), the hearing of the affected side (tumor side) of the patients with AN were as follows: normal hearing (PTA < 25dB HL) in 5 cases, mild loss (PTA 26-40 dB HL) in 12 cases, moderate loss (PTA 41-60 dB HL) in 9 cases, severe loss (PTA 61-80 dB HL) in 13 cases, profound loss (PTA > 81 dB HL) in 10 cases. Compared with the HC group, the cognitive function of AN patients with mild to profound hearing loss decreased in various degrees, see Table 7 and Table 8.

|  | Normal hearing (n = 5) | Mild loss  (n = 12) | Moderate loss  (n = 9) | Severe loss  (n = 13) | Profound loss  (n = 10) |
| --- | --- | --- | --- | --- | --- |
| visuospatial executive | -1.607(0.132) | -2.723(0.006)^**^ | -3.159(0.002)^**^ | -4.170 (*P* < 0.001)^***^ | -3.845(*P* < 0.001)^***^ |
| naming | -0.741(0.556) | -1.151(0.250) | -0.202(0.840) | -2.817(0.005)^**^ | -1.159(0.246) |
| attention | -1.022(0.488) | -1.583(0.113) | -1.713(0.087) | -4.174 (*P* < 0.001)^***^ | -4.482(*P* < 0.001)^***^ |
| language | -0.783(0.475) | -2.181(0.029)^*^ | -1.362(0.173) | -2.591(0.010)^**^ | -2.797(0.005)^**^ |
| language: Sentence repetition | -0.267(0.437) | -1.810(0.070) | -1.181(0.238) | -2.398(0.017)^*^ | -2.676(0.007)^**^ |
| language: fluency task | -0.267(0.959) | -2.582(0.010)^**^ | -3.053(0.002)^**^ | -3.327(0.001)^***^ | -2.873(0.004)^**^ |
| abstract thinking | -0.413(0.718) | -0.058(0.954) | -2.265(0.024)^*^ | -2.860(0.004)^**^ | -0.690(0.490) |
| delayed recall | -1.613(0.116) | -0.870(0.384) | -0.165(0.869) | -3.027(0.002)^**^ | -2.782(0.005)^**^ |
| orientation | -0.737(0.718) | -0.629(0.529) | -1.886(0.059) | -3.236(0.001)^***^ | -4.071(*P* < 0.001)^***^ |
| MoCA scores | -1.536(0.132) | -2.016(0.044)^*^ | -2.325(0.020)^*^ | -3.961(*P* < 0.001)^***^ | -3.651(*P* < 0.001)^***^ |

**Table 7 Comparison of MoCA among patients with different degrees of hearing loss and HC group**

Data are presented as Z values (*P* values). *Z* and p values were obtained by Mann-Whitney U nonparametric test. Normal hearing: PTA < 25dB HL; mild loss: PTA 26-40 dB HL; moderate loss: PTA 41-60 dB HL; severe loss: PTA 61-80 dB HL; profound loss: PTA > 81 dB HL. *, *P* < 0.05; **, *P*≤ 0.01; ***, *P* ≤ 0.001. N/A: not available.

**Table 8 Comparison of cognitive function among patients with different degrees of hearing loss and HC group**

|  | Normal hearing (n = 5) | Mild loss  (n = 12) | Moderate loss  (n = 9) | Severe loss  (n = 13) | Profound loss  (n = 10) |
| --- | --- | --- | --- | --- | --- |
| MoCA scores | -1.536(0.132) | -2.016(0.044)* | -2.325(0.020)* | -3.961(*P* < 0.001)*** | -3.651(*P* < 0.001)*** |
| RAVLT immediate recall | -0.893(0.389) | -2.869(0.004)** | -1.359(0.174) | -3.391(0.001)*** | -3.858(*P* < 0.001)*** |
| RAVLT delay recall | -1.228(0.230) | -2.117(0.034)* | -1.058(0.293)^a^ | -3.321(0.001)^a^*** | -4.177(*P* < 0.001)^a^*** |
| Stroop A (s) | -1.155(0.263) | -0.666(0.505) | -0.157(0.875) | -1.904(0.057) | -2.367(0.018)* |
| Stroop B (s) | -0.648(0.528) | -0.774(0.439) | -0.448(0.654) | -3.378(0.001)*** | -3.165(0.002)** |
| Stroop C (s) | -0.680(0.515) | -2.060(0.039)* | -1.837(0.066) | -2.572(0.010)** | -3.274(0.001)*** |
| SDMT | -1.349(0.183) | -0.794(0.427) | -1.034(0.301) | -2.664(0.008)** | -3.006(0.003)** |
| TMT A (s) | -0.754(0.462) | -1.116(0.265) | -1.351(0.177) | -3.265(0.001)*** | -3.202(0.001)*** |
| TMT B (s) | -0.074(0.943) | -1.995(0.046)* | -0.301(0.763) | -3.104(0.002)** | -2.431(0.015)* |

Data are presented as T or Z values (*P* values). *T* and ^a^ *P* values were obtained by t-test. Z values were obtained by Mann-Whitney U nonparametric test. Normal hearing: PTA < 25dB HL; mild loss: PTA 26-40 dB HL; moderate loss: PTA 41-60 dB HL; severe loss: PTA 61-80 dB HL; profound loss: PTA > 81 dB HL. *, *P* < 0.05; **, *P* ≤ 0.01; ***, *P* ≤ 0.001. N/A: not available.

**4. Results of correlation analysis between the course of disease and cognition**

The course of disease was negatively correlated with MoCA subscores on visuospatial executive and attention, RAVLT delayed recall, and SDMT, and positively correlated with Stroop A, B, HAMA, and the left-sided PTA of LAN. See Figure 1 and 2.


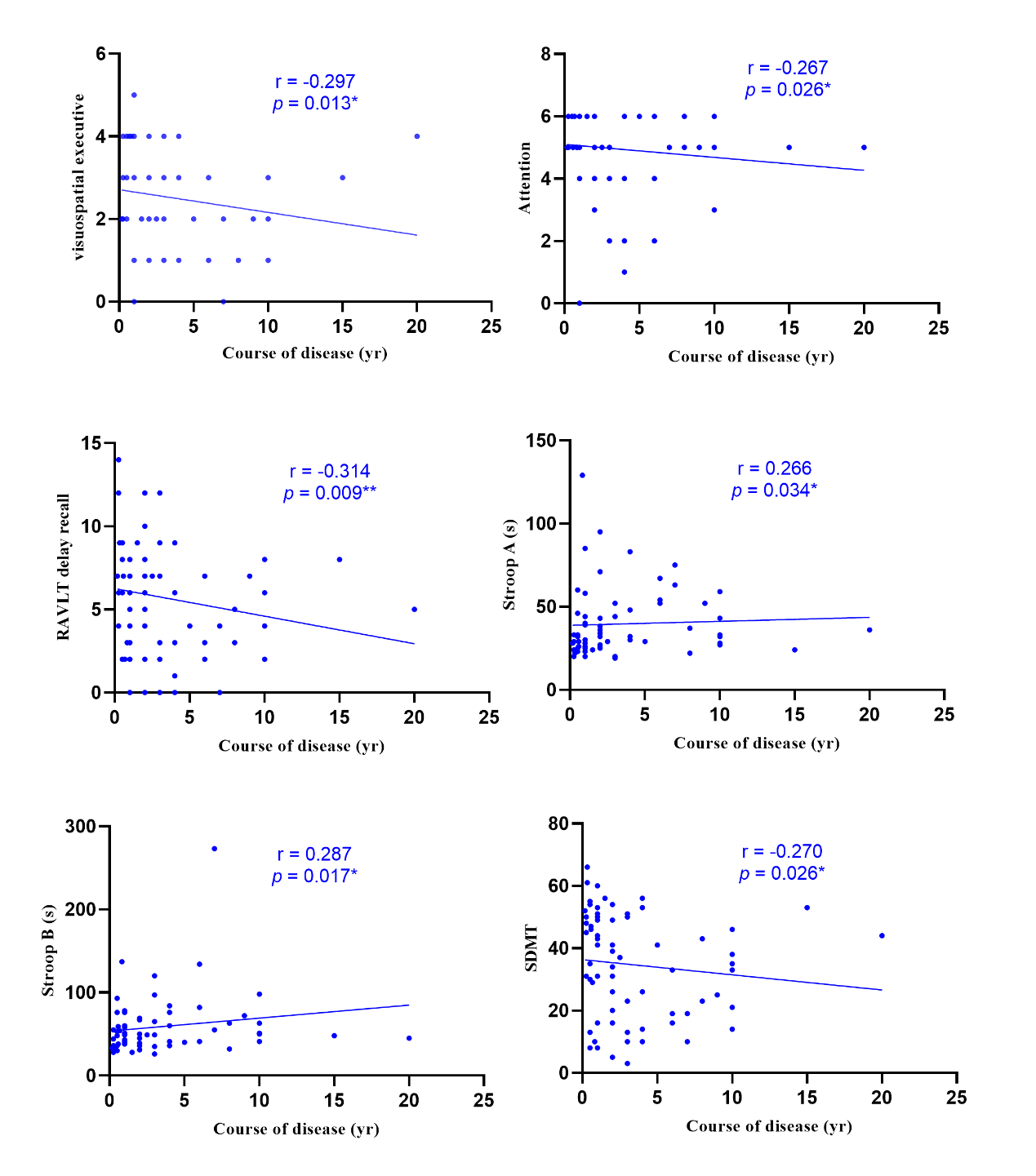


**Figure 1 correlation between course of disease and cognitive scale**

*, *P* < 0.05；**, *P* < 0.01

| 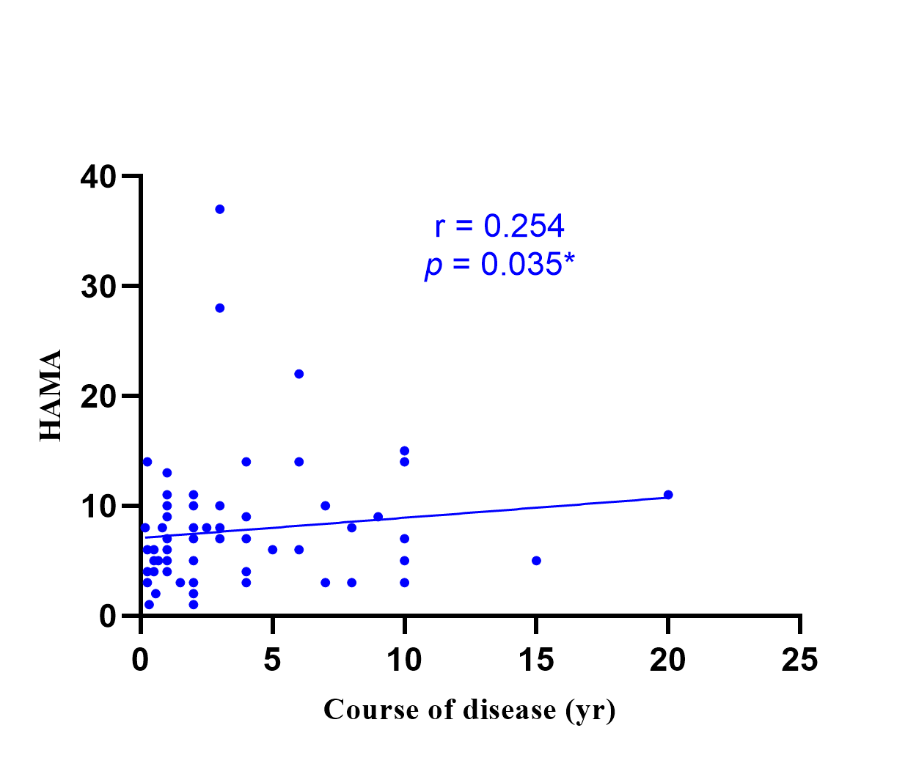 |
| --- |
| **Figure 2 Correlation between course of disease and HAMA**  *, *P* < 0.05；**, *P* < 0.01; HAMA: Hamilton anxiety scale  **5. Results of correlation analysis between node efficiency and cognition** |

The differential brain regions of node efficiency are shown in Supplementary Table 8, and Table 9. Spearman correlation analysis was performed between node efficiency of differential brain regions and cognitive performance. Widespread decreased node efficiency affected general cognitive function mainly in the frontal lobe, parietal lobe, insular, and limbic system, involving the default mode network (DMN), frontoparietal network, and salience network, which related to advanced cognition. The results are shown in Supplementary Table 9 and Table 10.

**Table 9** **Results of correlation analysis between node efficiency and cognition (1)**

| Differential nodes shared by LAN and RAN | MoCA | RAVLT immediate recall | RAVLT delay recall | SDMT |
| --- | --- | --- | --- | --- |
| Precentral_R | 0.249(0.003) | 0.210(0.013) | 0.227(0.007) | 0.156(0.067) |
| Frontal_Sup_R | 0.210(0.013) | 0.187(0.028) | 0.209(0.014) | 0.168(0.048) |
| Rolandic operculum R | 0.280(0.001) | 0.241(0.004) | 0.262(0.002) | 0.183(0.032) |
| Frontal_Inf_Oper_R | 0.190(0.025) | 0.173(0.041) | 0.199(0.019) | 0.142(0.096) |
| Insula_R | 0.248(0.003) | 0.207(0.015) | 0.209(0.014) | 0.178(0.037) |
| Frontal_Inf_Tri_R | 0.193(0.023) | 0.144(0.090) | 0.155(0.068) | 0.127(0.137) |
| Cingulum_Ant_R | 0.092(0.281) | 0.170(0.045) | 0.178(0.037) | 0.061(0.476) |
| Cuneus_R | 0.165(0.052) | 0.137(0.107) | 0.188(0.026) | 0.080(0.348) |
| Cingulum_Mid_R | 0.189(0.025) | 0.149(0.081) | 0.166(0.051) | 0.119(0.166) |
| Postcentral_R | 0.217(0.010) | 0.204(0.016) | 0.242(0.004) | 0.121(0.157) |
| Parietal_Inf_R | 0.172(0.043) | 0.128(0.133) | 0.184(0.030) | 0.082(0.338) |
| Precuneus_R | 0.198(0.020) | 0.184(0.030) | 0.202(0.017) | 0.104(0.226) |
| SupraMarginal_R | 0.265(0.002) | 0.231(0.006) | 0.287(0.001) | 0.158(0.064) |
| Parietal_Sup_R | 0.272(0.001) | 0.207(0.015) | 0.251(0.003) | 0.145(0.089) |
| Paracentral_Lobule_R | 0.157(0.062) | 0.165(0.053) | 0.249(0.003) | 0.114(0.183) |
| Differential nodes unique to LAN | MoCA | RAVLT immediate recall | RAVLT delay recall | SDMT |
| Lingual_R | 0.162(0.056) | 0.145(0.089) | 0.183(0.031) | 0.083(0.335) |
| Occipital_Sup_R | 0.195(0.022) | 0.175(0.040) | 0.175(0.039) | 0.110(0.200) |
| Occipital_Inf_R | 0.202(0.017) | 0.154(0.070) | 0.174(0.040) | 0.191(0.025) |
| Differential nodes unique to RAN | MoCA | RAVLT immediate recall | RAVLT delay recall | SDMT |
| Supp_Motor_Area_R | 0.149(0.081) | 0.158(0.063) | 0.206(0.015) | 0.144(0.093) |
| Cingulum_Post_R | 0.213(0.012) | 0.181(0.033) | 0.202(0.017) | 0.111(0.195) |
| Frontal_Sup_Medial_R | 0.202(0.017) | 0.218(0.010) | 0.224(0.008) | 0.215(0.011) |

Data are presented as Spearman correlation coefficient (r value) and (*P* value); LAN: left acoustic neuroma; RAN: right acoustic neuroma.

| Differential nodes shared by LAN and RAN | stroopA (s) | stroopB (s) | stroopC (s) | TMT A (s) | TMT B (s) |
| --- | --- | --- | --- | --- | --- |
| Precentral_R | -0.179(0.040) | -0.124(0.147) | -0.215(0.011) | -0.128(0.135) | -0.171(0.044) |
| Frontal_Sup_R | -0.238(0.006) | -0.162(0.057) | -0.200(0.019) | -0.112(0.190) | -0.144(0.090) |
| Rolandic operculum R | -0.181(0.037) | -0.206(0.015) | -0.247(0.003) | -0.159(0.061) | -0.200(0.018) |
| Frontal_Inf_Oper_R | -0.189(0.029) | -0.136(0.111) | -0.203(0.017) | -0.106(0.214) | -0.174(0.040) |
| Insula_R | -0.191(0.028) | -0.166(0.050) | -0.237(0.005) | -0.158(0.063) | -0.218(0.010) |
| Frontal_Inf_Oper_R | -0.184(0.034) | -0.176(0.038) | -0.205(0.016) | -0.111(0.192) | -0.187(0.028) |
| Hippocampus_R | -0.128(0.142) | -0.107(0.212) | -0.183(0.032) | -0.167(0.050) | -0.182(0.032) |
| Frontal_Mid_R | -0.104(0.233) | -0.022(0.797) | -0.022(0.796) | -0.212(0.012) | -0.114(0.180) |
| Cingulum_Mid_R | -0.158(0.070) | -0.063(0.461) | -0.190(0.025) | -0.087(0.310) | -0.140(0.101) |
| Postcentral_R | -0.114(0.191) | -0.127(0.136) | -0.188(0.027) | -0.054(0.525) | -0.087(0.309) |
| Parietal_Inf_R | -0.103(0.238) | -0.134(0.116) | -0.169(0.047) | -0.023(0.784) | -0.028(0.744) |
| Precuneus_R | -0.129(0.138) | -0.082(0.339) | -0.177(0.038) | -0.063(0.459) | -0.104(0.224) |
| SupraMarginal_R | -0.162(0.062) | -0.213(0.012) | -0.211(0.013) | -0.150(0.077) | -0.167(0.049) |
| Parietal_Sup_R | -0.171(0.049) | -0.139(0.102) | -0.233(0.006) | -0.121(0.156) | -0.126(0.141) |
| Paracentral_Lobule_R | -0.148(0.088) | -0.171(0.044) | -0.229(0.007) | -0.055(0.522) | -0.121(0.156) |
| Differential nodes unique to LAN | stroopA (s) | stroopB (s) | stroopC (s) | TMT A (s) | TMT B (s) |
| Occipital_Inf_R | -0.244(0.005) | -0.193(0.023) | -0.161(0.060) | -0.194(0.022) | -0.175(0.039) |
| Differential nodes unique to RAN | stroopA (s) | stroopB (s) | stroopC (s) | TMT A (s) | TMT B (s) |
| Supp_Motor_Area_R | -0.206(0.017) | -0.148(0.082) | -0.184(0.031) | -0.086(0.313) | -0.145(0.089) |
| Frontal_Sup_Medial_R | -0.226(0.009) | -0.177(0.037) | -0.254(0.003) | -0.196(0.021) | -0.212(0.012) |
| Cingulum_Post_R | -0.142(0.102) | -0.097(0.254) | -0.182(0.033) | -0.079(0.353) | -0.118(0.167) |
| Thalamus_R | -0.119(0.173) | -0.084(0.325) | -0.168(0.049) | -0.150(0.078) | -0.169(0.047) |

**Table 10** **Results of correlation analysis between node efficiency and cognition (2)**

Data are presented as Spearman correlation coefficient (r value) and (*P* value); LAN: left acoustic neuroma; RAN: right acoustic neuroma.

**6. Negative results of correlation analysis between clinical metrics and cognition**

Patients with acoustic neuroma are often complicated with tinnitus symptoms. In this study, AN patients accompanying tinnitus were assessed using the tinnitus handicap inventory (THI) scale^2^. Higher scores indicate greater severity and greater impact on daily life. Correlation analysis showed there was no significant correlation between THI and cognitive scale, or THI and anxiety, depression scale.

To explore whether tumor size affects cognitive function, we took the largest tumor diameter of the largest level of tumor on MRI as tumor size, and Spearman correlation analysis was performed between tumor diameter and cognitive function in each grade of Koos. The results showed that there was no significant correlation between tumor size and cognitive function.

**7. The results of local metrics of structural brain network were as follows:**

Node local efficiency: Compared with the HC group, significantly decreased node local efficiency in the LAN group was found in the right precuneus, right insula, right suprmarginal gyrus, left superior frontal gyrus. Right interior frontal gyrus. Right precentral gyrus, and right paracentral lobule; significantly decreased node local efficiency in the RAN group was found in the right precuneus, right insula, right suprmarginal gyrus, left superior frontal gyrus. Right interior frontal gyrus. Right precentral gyrus, and right paracentral lobule (*P* < 0.05, FDR corrected).

Node efficiency: Compared with the HC group, both left and right AN patients showed a more extensive decrease in the node efficiency of the brain region, mainly in the frontal lobe, occipital lobe, parietal lobe, limbic system, basal ganglia, thalamus, and so on. Only in the LAN group, the node efficiency of the left middle and inferior temporal gyrus increased, which may be due to the activation of compensation. The differential brain regions in node efficiency are shown in Table 8, and Table 9.

Node shortest path: Compared with the HC group, the shortest path of the LAN and RAN group increased in the right precentral gyrus, right inferior frontal gyrus, right central sulcus cover, right peritalar fissure cortex, and right postcentral gyrus (*P* < 0.05, FDR corrected).

Node clustering coefficient: Compared with the HC group, the node clustering coefficient decreased in the right lentiform putamen in the LAN group, and decreased in the left posterior cingulate gyrus, right hippocampus, right inferior parietal angular gyrus, right thalamus, and right inferior temporal gyrus in the RAN group (*P* < 0.05, FDR corrected).

Degree centrality: Compared with the HC group, the decreased brain regions of patients with LAN and RAN were mainly in bilateral lenticular putamen, right superior frontal gyrus, right central sulcus cover, right postcentral gyrus, right thalamus, right superior orbital frontal gyrus, right island, right hippocampus, right precuneus, right superior marginal gyrus, right globus pallidus, right amygdala, left superior parietal gyrus, left middle occipital gyrus and left angular gyrus. In addition, the degree centrality of the LAN decreased, while the RAN had no similar changes in these brain regions. The degree centrality of left paracentral lobule in RAN patients decreased in the bilateral precentral gyrus, hippocampus, and thalamus, right dorsolateral superior frontal gyrus, right superior parietal gyrus and inferior frontal gyrus, left middle temporal gyrus, and left posterior cingulate gyrus, but there was no similar change in left acoustic neuroma in these brain regions (*P* < 0.05, FDR corrected).


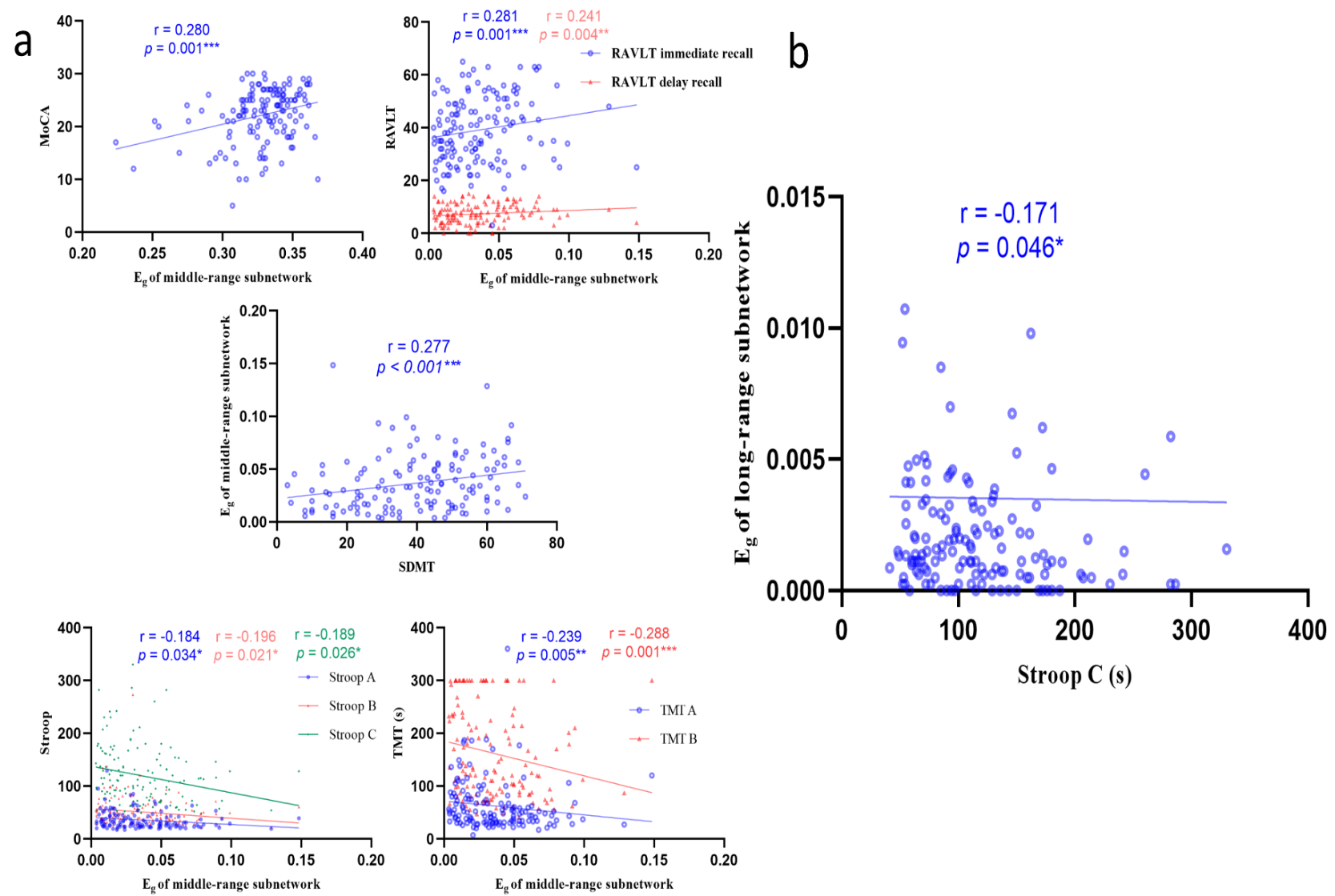
**8. Correlations between the global metrics (Eg, Eloc) of middle-range subnetwork and cognitive scale**

**Figure 3** Correlations between the global metrics (E_g_, E_loc_) of middle-range subnetwork and cognitive scale. **a** Correlations between the E_g_ of middle-range subnetwork and cognitive scale. The E_g_ of middle-range subnetwork was negatively correlated with cognition function (*n =* 139, *P* < 0.05). **b** Correlation between the E_g_ of long-range subnetwork and Stroop C. MOCA: Montreal cognitive assessment; RAVLT: Rey auditory verbal learning test; SDMT: symbol digit modalities test; Stroop: Stroop color-word test; TMT: trail-making test

**Discussion**

**The course of disease and cognition**

The course of disease was positively correlated with Stroop A and B, and negatively correlated with MoCA subscores on attention and visuospatial executive, RAVLT delayed recall, SDMT, indicating that with the development of course of disease, the ability of visuospatial executive, attention, memory, motor speed and information processing speed decrease gradually, so early intervention may delay the effect of disease on cognitive function. We found that patients with different grades of AN had different degrees of cognitive decline, however, there was no correlation between tumor size and cognitive function, demonstrating that tumor size was not the main factor for cognitive decline in AN patients. Meanwhile, this study also found that cognitive function decreased in patients with different degrees of hearing loss, and there was a significant correlation between PTA and cognitive function. It is suggested that hearing loss may have a greater effect on the cognitive function of patients with AN.

**Acoustic neuroma patients with tinnitus and cognition**

Patients with acoustic neuroma are often associated with tinnitus. In our research, those patients accounted for 43.5% (30/69). To determine whether tinnitus interfered with the experiment, we tested the THI in patients with tinnitus symptoms and analyzed the correlation with neuropsychological tests and THI scores. The results showed that tinnitus did not affect cognitive function, anxiety, and depression, however, Chen et al.^3^ found that tinnitus affects cognitive function. We analyze the possible reasons as follows: first, the severity of tinnitus patients in two experiments are different. Most of the tinnitus patients in their study are moderate and severe tinnitus of grade 3-4 (25/35, 71.43%), while most of our patients are mild tinnitus of grade 1-2 (29/30, 96.67%), and only 3.33% of moderate and severe tinnitus patients (1/30). Therefore, it may cause patient selection bias. The tinnitus symptoms of patients in our study are generally mild, which may be because patients are often not only complicated with symptoms such as tinnitus and hearing loss, but also headache, dizziness, and unstable walking, so it is more likely to attract the attention of patients and doctors. then examination found the existence of acoustic neuroma. Second, the heterogeneity of patients, our cases were acoustic neuroma with tinnitus symptoms, their cases were patients with pure right tinnitus, so it may cause differences in experimental results. Third, the statistical power was limited due to the small number of tinnitus patients in the group, which also may have an impact on the experimental results. Meanwhile, Chen's study also found no correlation between tinnitus and anxiety or depression scale, which is consistent with our findings.

**The relation between local metrics and cognitive function**

SDMT, Stroop, and TMT tests evaluate attention, executive function, working memory, motor speed, and so on. The brain regions related to SDMT, Stroop, and TMT were mainly located in frontal lobe, insular, parietal lobe, limbic system (including hippocampus, cingulate gyrus), and precuneus (Supplementary Table 9 and Table 10). The decrease of node efficiency in these brain regions was closely related to cognitive impairment. The prefrontal cortex is an important region of executive control, and it is also highly related to working memory. FAN et al.^4^ used dynamic causal modeling (DCM) analysis to confirm that conflict processing (such as Stroop test) was linked to the integration of cingulate gyrus, prefrontal cortex, and motor area. The insular controls the generation and origin of the senses and emotions, and is the core region to salience network, and mainly plays a key role in the switching between cognitive function-related networks (such as the DMN and the dorsal attention network)^5^. Hippocampus is associated with memory^6^. The precuneus and cingulate cortex are core DMN nodes that play an important role in higher-order cognitive functions such as episodic memory^7^. Therefore, the brain regions with decreased node efficiency affect executive function, memory, attention, and other advanced cognitive functions, which may be the reason for the decline of patients' cognitive function.

**References**

1. Koos, W., Spetzler, R. & Lang, J. *Color Atlas of Microneurosurgery*. (Stuttgart：Thieme Germany, 1993).

2. Newman, C., Jacobson, G. & Spitzer, J. Development of the Tinnitus Handicap Inventory. *Archives of otolaryngology--head & neck surgery* **122**, 143-148, doi:10.1001/archotol.1996.01890140029007 (1996).

3. Chen, Y.-C. et al. Alterations of the default mode network and cognitive impairment in patients with unilateral chronic tinnitus. Quantitative Imaging in Medicine and Surgery 8, 1020-1029, doi:10.21037/qims.2018.11.04 (2018)

4. Fan J, Hof P, Guise K, Fossella J, Posner M. The functional integration of the anterior cingulate cortex during conflict processing. Cerebral cortex (New York, NY : 1991) 2008;18:796-805.

5. Sidlauskaite J, Sonuga-Barke E, Roeyers H, Wiersema J. Altered intrinsic organisation of brain networks implicated in attentional processes in adult attention-deficit/hyperactivity disorder: a resting-state study of attention, default mode and salience network connectivity. European archives of psychiatry and clinical neuroscience 2016;266:349-357.

6. Lisman J, Buzsáki G, Eichenbaum H, Nadel L, Ranganath C, Redish A. Viewpoints: how the hippocampus contributes to memory, navigation and cognition. Nature neuroscience 2017;20:1434-1447.

7. Buckner R, DiNicola L. The brain's default network: updated anatomy, physiology and evolving insights. Nature reviews Neuroscience 2019;20:593-608.
